# Ectopic Gestation and Pelvic Hematocele

**Published:** 1889-03

**Authors:** 


BUFFALO MEDICAL AND SURGICAL JOURNAL

A MONTHLY REVIEW OF MEDICINE AND SURGERY.

EDITORS:

THOS. LOTHROP, M. D., - - - W. W. POTTER, M. D.

All communications, whether of a literary or business character, should be addressed to the editors: 284 FRANKLIN STREET, BUFFALO, N. Y.

### Editorial.

### *ECTOPIC GESTATION, AND PELVIC HEMATOCELE,*

CONSIDERED IN THE LIGHT OF MR. TAIT'S VIEWS—BEING PRINCIPALLY A DISCUSSION OF THESE AS ENUNCIATED IN HIS LECTURES ON THIS SUBJECT.[1]

In the light of his achievements in the surgery of ectopic gestation, no future consideration of this subject can be approached without due acknowledgment of the value of Mr. Tait's contributions toward the clearing up of doubtful questions of this hitherto much discussed, but little understood, *bête noir* of abdominal surgery.

The lectures, which are taken as the text of these remarks—chiefly in the sense of a review—are the most important addition to the literature of the subject since the appearance of Bernutz and Goupil's Memoirs. This book, as Mr. Tait most justly remarks, has received by far too little notice, containing, as it does, a basis scientifically correct for the pathological study of aberrant gestation; while, on the other hand, from the point of treatment, it is absolutely of no value whatever, barely suggesting, as it will be found, the possibility of the success of surgical interference. In this connection, it is interesting to note that the passage of the electric current through the fetal sac is suggested by these authors as a vague possibility of benefit. The use of this

1. Lectures on Ectopic Pregnancy and Pelvic Hematocele. By Lawson Tait, F. R. C. S. Edin. and Eng., LL. D., Prof. Gynecology in Queen's College in Birmingham, etc., etc., etc.

agent in extra-uterine pregnancy will be referred to further on. Mr. Tait's views as to the pathological changes in the tubes, the retention of the ova thus being made possible when impregnated, though not capable of exact demonstration, is at once so plausible and natural, that it impresses the careful thinker as perhaps the best possible hypothesis of the first step of this most dread surgical affection in women.

Mr. Tait regards all ectopic pregnancies as primarily tubal, admitting, however, the possibility of an ovarian pregnancy. The time of rupture is placed before the fourteenth week. Bernutz and Goupil extend the period to four months. Mr. Tait's opinion on this point is more likely to be correct. His view of the history of the rupture is as follows:

"It takes two directions (*a*) into the peritoneum, which is the fatal form; and (*b*) into the cavity of the broad ligament, a form which yields the variety of ectopic gestation, which I propose to call the extra-peritoneal which was called the sous-peritoneo-pelvienne variety by Dezeimeris, and which alone yields all the cases which go on to the period of viability, all the lithopedia, all the suppurating cysts discharging into bladder, rectum, etc., and also the cases which by *secondary rupture* of the *ovum cyst*, get called abdominal pregnancy."

Mr. Tait first proposed this view of the varieties of ectopic gestation in 1873. Its simplicity, and the logical defense of his position in expounding his theory, render it probable that it will ultimately be accepted by all. Mr. Tait's "genealogical" table of ectopic gestation is as follows:

I.—Ovarian, possible but not yet proved.

II.—Tubal, in free part of tube, is (*a*) contained in tube up to fourteenth week, at or before which time *primary* rupture occurs, and then the progress of gestation is directed into

(*b*) Abdominal or intra-peritoneal gestation uniformly fatal (unless removed by abdominal section), primarily by hemorrhage, secondarily by suppuration of the sac and peritonitis;

(*c*) Broad ligament or extra-peritoneal gestation;

(*d*) may develop in broad ligament to full time and be removed at viable period as living child;

(*e*) may die and be absorbed as extra-peritoneal hematocele;

(*f*) may die and the suppurating ovum may be discharged at or near umbilicus, or through bladder, vagina, or intestinal tract;

(*g*) may remain quiescent as lithopedion;

(*h*) may become abdominal or intra-peritoneal gestation by secondary rupture.

III.—Tubo-uterine or interstitial is contained in part of tube embraced by uterine tissue, and, so far as is known, is uniformly fatal by primary intra-peritoneal rupture (as *b*) before fifth month.

As has already been stated, Mr. Tait does not deny the possibility of ovarian pregnancy, but doubts its probability. All the cases of this variety of pregnancy, save Spiegelberg's, he regards as questionable, or entirely fallacious.

As to the diagnosis of ectopic gestation previous to the period of rupture, Mr. Tait plainly states that nothing positive can be insisted upon. That is to say, that while it may be held and proven by operation that ectopic gestation has existed, still in other cases, where the diagnosis is just as positive with just as strong presumptive evidence, an error in diagnosis will be found to have been made, and no ectopic pregnancy exists. This view comes with peculiar force in view of the author's vast experience. He cites one case in which the lesion occurred a second time in the same woman, who, in spite of her own previous experience, did not recognize or suspect her condition, and died of hemorrhage from rupture. Than this nothing can be stronger proof that symptoms may count for naught, and that while it is possible to categorize upon the presence of this symptom and that, as a proof of extra-uterine gestation, one or many or all of these symptoms may be absent and the only presage of the condition will be the rupture. In this connection it is fitting to refer to two sets of cases, almost unique in their persistent claim for record as remarkable diagnoses. As will be seen, the claims of the one, treated by electricity, are presumptive, proving nothing. Those of the other are presumptuous and pertinacious. The cases referred to are those of Dr. J. G. Allen, of Philadelphia, *American Journal of Obstetrics*, vol. v., p. 161, 1872, and Dr. H. A. Kelly, reported in the *American Journal of Obstetrics*, Aug., 1886.

The cases of Dr. Allen are frequently referred to, especially in reference to the treatment of this condition by the electric current. It is not our purpose to discuss treatment at this point, but to consider diagnosis simply. In the first of Dr. Allen's cases, something resembling a decidua was passed at about the second month of pregnancy. Later, ballottement gave assurance of the presence of a fetus. The current being passed, the tumor ceased growing and gradually diminished to the size of a large fist. In Dr. Allen's second case the history is defective. No symptoms were discoverable, and all that is asserted as proven is that the tumor decreased in size after the application of the current. In his third case, which the after-history shows indisputably, by the discharge of the fetal bones, to have been extra-uterine pregnancy, the condition was not even suspected. Nothing abnormal was noticed till after the sixth month. The woman had supposed herself pregnant, and engaged Dr. Allen to attend her. "A special examination," by which a tumor could be detected, led him to think it a fibroid growth. Here, then, are three cases considered extra-uterine. One has proven, by its termination, its right to be so maintained. In it, the condition was not even suspected. The other two are, if not supposititious, at least suppositional; and this statement is true of all cases claimed to be extra-uterine fetation on no better evidence than some or many of the symptoms of pregnancy, the subsequent passage of the electric current, and the after-shrinkage of the tumor. Such a history cannot be claimed to be distinctly peculiar to this condition. At best, it is the expression only of a probability. It is not even presumptive evidence. Then, again, shrinkage of the cyst, as a pathognomonic symptom of extra-uterine fetation, would have no value, where diagnosis for operation by the abdominal section might be considered; shrinkage could only occur after the death of the fetus, and as advocates of the electric current desire only to kill it, operation from their standpoint would be a useless procedure. We will refer to this point further on. The case of Dr. Kelly is only worthy of attention as showing how a diagnosis may grow after operation, and points be developed by time as vital, that have no place in the original communication.

The special point since claimed in this case, is the shrinkage of the cyst. This step in the diagnosis is absolutely wanting in the original report of the case, and, therefore, is an after-thought, making its addition to a consideration of the subject simply superfluous, and therefore valueless. Nay, more; it is misleading and fallacious, in that it seeks to put up for a positive sign what previously had not been considered, but also loses sight of the fact, that as a diagnostic sign, where operation is held under advisal previous to rupture, it is positively of no value whatever. Further, the remarks of Dr. R. P. Harris, after the presentation of the case, distinctly say:

"The case reported by Dr. Kelly *had as clear a history as we ever find in the very early history of ectopic gestation, when it cannot be claimed that a positive diagnosis can be made.*"

Here is an admission by Harris himself, which we have taken the liberty to italicise, that a claimed positive diagnosis was only a guess, and that on mere presumptive evidence. Further, the passage of a piece of flesh, which the doctor did not see, is taken as a step in the diagnosis. This is in all cases questionable. First, because even in cases of undisputed ectopic gestation, the decidua is frequently, if not always, passed as shreds or debris, and attracts no attention whatever. If the membrane, then, is an important point in diagnosis, it must in these cases be wanting, and a decision reached without it. Further, in closing his discussion, Dr. Kelly reported having another "case of extra-uterine fetation on hand now, and is waiting for the death of the fetus, when he will operate." Where is the report of this case? There is certainly none on record.

One other case remains to be noticed, especially in reference to the use of the electric current, after a supposedly correct diagnosis. Dr. Wylie, *American Journal of Obstetrics*, vol. xix., p. 75, reports a "case of hydrosalpinx, diagnosticated as extra-uterine pregnancy."

The case had been first seen by Dr. Mann, of this city, who judged it extra-uterine pregnancy, passed the electric current at intervals for ten days, and sent the patient home. Dr. Wylie operated, as stated above. The question now arises whether many of the so-called early diagnoses, insisted upon by Dr. Thomas, are not merely errors, which are buried by the disappearance of the patient, who subsequently falls into the hands of a less confident diagnostician, which latter more correctly finds her trouble by operation, and removes it without trifling with it. Dr. Thomas says:

"Were I asked to what I attribute the good results obtained in my cases, I should feel forced, in simple candor, to run the risk of the charge of egotism, and to reply, 'I attribute it to these influences: first, early and positive diagnosis; second, the prompt resort to destruction of the life of the fetus during the early months (of twelve thus treated all recovered); and third, to an equally prompt resort to surgery in the later.'"

It is well to remark that what Mr. Tait places first—operation—Dr. Thomas places last. Mr. Tait insists upon the essential guess-work of early diagnosis, while Dr. Thomas insists upon its certainty. It would be interesting to know in what proportion of cases operated upon the diagnosis was verified, and in what proportion it failed. It is significant that, after the above positive claim for early diagnostic precision, Dr. Thomas should write:

"After all that has been said with regard to the diagnosis of ectopic gestation, it must be added that a positive conclusion is very generally difficult, and often impossible."

Why not confess that the diagnosis is, after all, only a suspicion? If Dr. Thomas, with all his experience, writes the above, what must be thought of the claims of those whose diagnostic experience is based upon one or two cases, and who yet urgently, in season and out, herald their unprecedented feat of early diagnosis in this condition? In fact, if not in expression, Dr. Thomas and Mr. Tait are nearer together than, at first sight, would appear.

On the treatment after the rupture of the sac, Mr. Tait is unequivocally decided upon the propriety of operative interference. Dr. Thomas recites one case "trusted to nature," with ultimate recovery. With Mr. Tait's wonderful record, the weight of authority is on the side of early interference after rupture, or before if discovered. On what Dr. Thomas has to say upon the treatment of the sac before rupture, hinges its entire value as regards the question of correct diagnosis. In view of the uncertainty of this, to positive surgeons of the Tait type his dictum will have little weight. With these we believe that in competent hands, in case of doubt, the exploratory incision should be resorted to, and if the diagnosis is verified, the sac should be dealt with surgically, as hereafter to be noticed. The use of the electric current is all the less to be recommended in the light of Mr, Tait's successes. At best it only attempts the killing of the fetus, and can promise nothing more. It remains for the reproach of this treatment, that the sac and its contents still remain, and may at any subsequent period menace the life and safety of the patient. Or, in the most favorable event of suppuration and discharge, the comfort of the patient is for an indefinite period interfered with. The treatment appeals more to the electrical experimentist than to the practical surgeon—more to the trier of novelties than to him who is satisfied with nothing less than tangible results.

Mr. Tait's view that, in most instances, the so-called hematocele is a ruptured extra-uterine cyst, is fully substantiated, both by his investigations and those of Bernutz, to whom he gives especial credit.

Bernutz's classification of the possible varieties of intra-pelvic hemorrhage is worthy at this time of great consideration, and is:

1. Hemorrhage caused by the rupture of dilated utero-ovarian veins. (This kind of hematocele occurring sometimes in extra-uterine pregnancy, may be said to represent one of the varieties of thrombus in normal gestation.)

2. Hemorrhage caused by the rupture of the ovary. (This we see happen in cases of pregnancy, whether the product of conception occupies the uterus or not.)

3. Hemorrhage caused by the rupture of the Fallopian tube.

4. Hemorrhage from the fetal cyst itself having ruptured. (The largest number of cases fall under this last head; both it and the next are of special interest, because they are peculiar to extra-uterine pregnancy, while the three former belong also to intra-uterine pregnancy.)

5. Hemorrhage within the fetal cyst; (which may end in death without effusion of blood into the peritoneal cavity, and, therefore, may not produce a real hematocele.)

Had these views received the attention they deserve, there would not now be so much conflicting statement concerning the affection, or rather the symptoms they elucidate. Of hematocele due to suppressed menstruation, Bernutz also makes distinct mention. Of this variety Mr. Tait says:

"There are only two causes known to me, one very common, and one relatively rare. The first is sudden arrest of metrotaxis, which may be either normal menstruation, or the pseudo-menstruation which occurs so constantly after abdominal operations." . . .

He then goes on to elucidate this condition. The abdominal surgeon who has not studied or considered it, will find much information, and with it much comfort concerning a matter which must often have worried and perplexed him. The second cause of extra-peritoneal hematocele is effusion of blood into the broad ligament from ruptured tubal pregnancy of about the twelfth week. Of this Mr. Tait says it is "much more rare and probably much more fatal, certainly much more serious."

The scheme Mr. Tait has given of extra-uterine pregnancy so well speaks for itself, that there is no need of repeating his argument. It is perfectly plain that, if the primary rupture takes place so as to fall within the folds of the broad ligament, the hemorrhage must be limited. It is also perfectly clear that a further secondary rupture may occur into the peritoneal cavity, and will then be almost necessarily fatal, unless by the merest chance.

Mr. Tait, in considering the question of rupture into the broad ligament, clearly shows that no two cases necessarily have the same history. Some terminate quickly by the death of the fetus and rapid absorption, others go on in their development for varying length of time, then to die and suppurate, or remain quiescent as lithopedia. Still others may go on until full development, necessitating surgical interference; while others, as has been shown, rupture, and unless promptly dealt with, in the vast majority of instances, terminate fatally.

Of suppurating cysts, Mr. Tait, after calling attention to their various paths of exit—umbilicus, rectum, vagina and bladder—thus sums up:

"In all of these the history helps but little, for the story is seldom more than that of obscure pelvic trouble, ending in abscess, bursting and continually discharging into the rectum, and it is not till the arrest of some sharp spicula of fetal bone in the anus declares the true solution, that the nature of the case is discovered. . . . . The mortality is doubtless quite what is asserted by Parry, though I never saw a fatal case. All that have come under my own care have been easily cured by the complete emptying of the sac."

Of the fatality of cases opening into the bladder, Mr. Tait agrees with Parry, that they are much more fatal than those opening in the other directions. Mr. Tait has never seen one of these cases in its early stages, but expresses the belief that the correct method of dealing with them is by abdominal section. He says:

"I feel quite confident that if these cases were dealt with by opening from above in their earlier stages, much of their mortality would disappear, and the patients would be spared years of suffering. I would treat them as I do pelvic abscesses, and if the peritoneum were opened I should close it in my usual fashion, by stitching the opening in the walls of the cavity of the broad ligament to the opening in the parietal peritoneum, after emptying the decomposing debris and cleaning out the cavity. I have now done over fifty operations of this nature, and not only has there been no mortality, but the cures have been so rapid, complete, and permanent, as to give me perhaps more satisfaction than almost any other class of my work."

For a complete discussion of what Mr. Tait terms the minority of the minority of cases, *i. e.*, where the ovum survives and grows toward full time, the reader must follow minutely the argument of the author. To do it full justice, almost a complete reproduction would be necessary. Jessop's remarkable case is cited at length, as the only instance of the so-called "abdominal" pregnancy on record. Mr. Tait, rightly we agree, refuses to accept those collected by Parry. The difficulty of diagnosis, in broad-ligament cases, approaching maturity, is thoroughly discussed. Conditions conducing to error are, extreme thinness of the uterine walls in normal pregnancy, displacement of the normally pregnant uterus, during the early months of pregnancy, complicated with fibro-myoma or cystic diseases of the uterus, and more rarely pregnancy in one-half of a double uterus. The greatest difficulties in diagnosis are met, according to Mr. Tait's experience, after the death of the child, or, at least, when the time of the expected confinement has passed so long that if there is a child, it is sure to be dead. "No history," says Mr. Tait, "however complete, is of sufficient weight to establish a diagnosis, unless there be some distinct physical signs in support of it." Quoting from Campbell's curious work, referred to by Ramsbotham as "a publication full of most valuable facts and deep research," he cites the following passage, showing how little trust can be placed in histories :

"In many instances of the different varieties of misplaced gestation, the catamenia are suspended; frequently, however, they appear regularly in each of the early months; in some cases they flow at uncertain periods; and in other examples they are either profuse or limited in quantity. In many cases, at an uncertain period of gestation, we have hemorrhage, uterine effusions, the extrusion of coagula, of bodies which resemble moles, or portions of placenta. These appearances have occasionally led to the belief that the patient has aborted, so that the ovum was originally not extra- but intra-uterine, and had escaped through a rent in the uterus into the peritoneal cavity, the extruded body, in either case, being viewed as the placenta. Cases attended with much uterine excitement, whether arising from unusual exertion, or some external injury, are most likely to be accompanied by these later phenomena."

Two symptoms are regarded by Mr. Tait as invariable in extra-uterine gestation which has gone past the period : a "show"

during false labor, and a diminution of size after false labor. This opinion is fortified by Parry's authority, which is regarded throughout as especially weighty.

As to the treatment of these extra-uterine fetal cysts, Mr. Tait leaves no doubt or question as to his position. Tapping is condemned, as a diagnostic procedure. The author says: "I open the abdomen and make out the condition." The use of the trocar in diagnosis, as shown by Parry, is simply a record of mortality, and cannot be too strongly condemned. The treatment by electricity, at or near full time, in order to kill the child when viable, is condemned by Mr. Tait in the strongest terms, especially as its death does not bring safety to the mother. His own experience has shown that, by operation, both may be saved, and his argument cannot be refuted. The treatment by electricity has been referred to before. Mr. Tait has no place for it. Dr. Buckmaster's case is cited at length. Mr. Tait here clearly has the best of the argument, and sums up his belief as follows:

"It is by no means clear from experience which we have had in this method, that the current is without harm, whether the diagnosis be correct or not, and it is equally without proof that it is sufficient to produce the effect desired."

In reply to the assertion that it is sometimes impossible to complete these operations, the following strong statement is made: "The rule ought to be that all such operations should be completed, and any man who has such want of skill and pluck as to stop in the middle of one of them, ought not to attempt them. THEY CAN ALL BE COMPLETED." It may be well to give the author's concluding creed in reference to the operation to save both mother and child:

He says, "I, therefore, advocate the principle of saving a child who has survived the catastrophe of the primary rupture of the tube by being extruded into the broad ligament. If its existence is recognized during life, the mother ought to be carefully watched till the false labor sets in, just as we watch for a case of puerperal hysterectomy and seize the onset of labor or its early stage, as the most favorable time for both mother and child. From this point of view, therefore, neither the time selected nor the details of the proceeding will be influenced save by two considerations: not to operate before the child is likely to be viable, provided the delay necessary does not prejudice the mother; and not to delay at all after the death of the child. I specially lay this down for the purpose, amongst others, of excluding all operations for the removal of the child by vaginal section." Mr. Tait then goes on to show the utter unjustifiableness of the vaginal section as a surgical procedure, and concludes, "I shall never, under any circumstances whatever, attack a sub-peritoneal pregnancy from the vagina."

Strong expression, indeed, but a weaker would be inadequate condemnation of a procedure which has its origin in timidity, and its end in failure.

The last portion of the book is devoted to a consideration of the pathological post-mortem researches of Hart and Carter. These are worthy the most careful study. By their frozen sections the peculiar arrangement of the peritoneum in extra-peritoneal ectopic gestation is fully elucidated, showing why, as Mr. Tait holds, the incision for operation should not be made in the middle line, so as to avoid opening the peritoneum. "In fact," he says, "the operation should not be an abdominal section at all, in the strict sense of the definition I have adopted."

As to the method of dealing with the placenta, Mr. Tait's conclusions are:

"I am, therefore, disposed for the present, at least, and until I am corrected by future experience, to advise in dealing with an ectopic gestation in the advanced stages, that we should deal with the fetus only, should empty the placenta of blood, and close the wound hermetically upon it."

The lectures are closed by a reference and tables of the length of time of retention of the fetus, and cases of lithopedion. More than simple reference to these is impossible.

Mr. Tait's tribute to Parry in the beginning of his book, and his continual citation throughout his discussion is, while deserved, generous and graceful, a respect from the living teacher to the voiceless but speaking dead.

No attempt has been made in this review to give the literature of the subject. With Mr. Tait's treatment of extra-uterine gestation, there is little need or use for ancient literature, save as it may be necessary to show how little was really known concerning it pathologically; *practically* nothing.

His treatment marks a new era in the handling of this condition, and may be taken as a starting point of its real surgery.

